# Age-dependent differences and similarities in the plasma proteomic signature of postoperative delirium

**DOI:** 10.1038/s41598-023-34447-7

**Published:** 2023-05-08

**Authors:** Rachel L. Oren, Erin J. Kim, Anna K. Leonard, Bernard Rosner, Lori B. Chibnik, Sudeshna Das, Francine Grodstein, Gregory Crosby, Deborah J. Culley

**Affiliations:** 1 Department of Anesthesiology, Perioperative and Pain Medicine, Harvard Medical School, Brigham and Women’s Hospital, 75 Francis St, Boston, MA 02115 USA; 2 Harvard Medical School, Brigham and Women’s Hospital, Boston, MA USA; 3 Department of Epidemiology, Harvard T.H. Chan School of Public Health, Boston, MA USA; 4 Department of Neurology, Harvard Medical School, Massachusetts General Hospital, Boston, MA USA; 5 Rush Alzheimer’s Disease Center, Rush University Medical Center, Chicago, IL USA; 6 Department of Anesthesiology and Critical Care, Perelman School of Medicine, University of Pennsylvania, Philadelphia, PA USA; 7 Present Address: Department of Neuroscience, Yale University, New Haven, CT USA; 8 Present Address: Department of Neurology, Perelman School of Medicine, University of Pennsylvania, Philadelphia, PA USA; 9 Present Address: Perelman School of Medicine, University of Pennsylvania, Philadelphia, PA USA

**Keywords:** Proteomic analysis, Biomarkers, Neurological manifestations, Disease prevention, Geriatrics, Neurodegeneration, Neurodegenerative diseases

## Abstract

Delirium is an acute confusional state and a common postoperative morbidity. Prevalent in older adults, delirium occurs at other ages but it is unclear whether the pathophysiology and biomarkers for the condition are independent of age. We quantified expression of 273 plasma proteins involved in inflammation and cardiovascular or neurologic conditions in 34 middle-aged and 42 older patients before and one day after elective spine surgery. Delirium was identified by the 3D-CAM and comprehensive chart review. Protein expression was measure by Proximity Extension Assay and results were analyzed by logistic regression, gene set enrichment, and protein–protein interactions. Twenty-two patients developed delirium postoperatively (14 older; 8 middle-aged) and 89 proteins in pre- or 1-day postoperative plasma were associated with delirium. A few proteins (IL-8, LTBR, TNF-R2 postoperatively; IL-8, IL-6, LIF, ASGR1 by pre- to postoperative change) and 12 networks were common to delirium in both age groups. However, there were marked differences in the delirium proteome by age; older patients had many more delirium-associated proteins and pathways than middle-aged subjects even though both had the same clinical syndrome. Therefore, there are age-dependent similarities and differences in the plasma proteomic signature of postoperative delirium, which may signify age differences in pathogenesis of the syndrome.

## Introduction

Delirium is an acute confusional state and one of the most common postoperative morbidities in aging adults^1–3^. It occurs in 15–45% of such patients after surgery and foreshadows serious consequences including prolonged hospital stay, discharge to a post-acute care facility, poor quality of life, accelerated decline to dementia, and higher 1-year mortality^4^. Though there are well-known predisposing factors such as advanced age, preoperative cognitive impairment, and frailty^5^, the mechanisms are not known and there are no well-validated biomarkers, so the ability to predict and prevent postoperative delirium is limited.

There have been many efforts to identify delirium biomarkers in blood and the list of proteins with the potential to predict the syndrome is growing. Putative delirium-associated proteins are involved in multiple biological systems and pathways including neurological and vascular function and disease, circadian biology, oxidative stress, and energy metabolism^6–8^, but some of the strongest candidates to date are proteins involved in inflammation and immune system regulation. Notable examples include C-reactive protein (CRP), interleukin (IL)-6, IL-8, and chitinase-3-like protein1 (CHI3L1)^9–14^ and recent large-scale analyses of the plasma proteome have identified others^12,13^. As such, although its pathogenesis is likely to be multifactorial, inflammation has emerged as one of the most prominent theories of post-surgical delirium^6,7,15^. Indeed, surgery initiates a robust aseptic inflammatory cascade of pro- and anti-inflammatory responses^16–18^ that is vital for repairing and restoring tissue homeostasis and ensuring normal recovery. However, the inflammatory response becomes dysregulated with age^19–26^ and dysfunction in this vital system is believed to contribute to the higher prevalence of postoperative delirium in older surgical patients. But postoperative delirium occurs across the age spectrum, albeit less commonly, including in middle aged persons before age-related changes in the immune, vascular, and neurologic systems set in. Different age groups have comparable clinical phenotypes of postoperative delirium and share some key symptoms but it is not known whether the pathophysiology and predictive biomarkers for the syndrome are similar irrespective of age because no investigations have compared the plasma proteome of delirium in different age groups. This question is of scientific and practical importance because similarities between ages would suggest the disorder has a common pathophysiology whereas divergence would imply the molecular correlates and biomarkers of the symptom complex differ with age. We hypothesized that the plasma protein signature of postoperative delirium is independent of age and investigated that possibility using a curated panel of proteins in middle-aged (45–60 years) and older (≥ 70 years) patients that developed delirium after elective spine surgery.

## Results

There were no statistically significant differences in sex, education, or 6-month mortality between subjects included in the proteomic analyses and those excluded due to lack of an available plasma sample but delirium was more common in the former group (Table 1). Of the subjects included in the analysis, there were no differences between the older and middle-aged groups in weight, BMI, sex, education, frailty status, or cognitive performance but the older group had more invasive surgeries and longer hospital stays. The dura was torn and repaired in 3 subjects (1 middle-aged, 2 old); only 1 (an old subject) became delirious. Overall, 28·9% (N = 22) of patients developed postoperative delirium; it was more common in older (33·3%; N = 14) than middle-aged patients (23·5%; N = 8) but the difference was not statistically significant (P = 0·35). Older patients with delirium were slightly older than those without (78 ± 4 vs 75 ± 4; P = 0.027) and had worse preoperative cognition by MiniCog (P = 0.052) and verbal fluency (P = 0.038) whereas educational attainment, frailty status, and surgery invasiveness and duration were similar (Table 2). In contrast, there were no such differences between middle-aged subjects with and without delirium (Table 2). In both age groups, as anticipated, delirium was associated with a longer length of hospital stay.

**Table 1:** Characteristics of patients included and excluded in proteomic analysis. p-values represent a comparison of differences between included middle-aged and older subjects.

Characteristic	Included				Excluded		
	Full sample (N = 76)	Older (N = 42)	Middle-aged (N = 34)	P-value	Full sample (N = 124)	Older (N = 58)	Middle-aged (N = 66)
Age, years	68 ± 11	77 ± 4	56 ± 3	0.000	64 ± 13	77 ± 4	54 ± 5
Weight, kg	82.5 ± 16.4	81.1 ± 14.6	84.2 ± 18.4	0.419	89.7 ± 22.0	85.8 ± 17.8	93.4 ± 24.9
Body Mass Index, kg/m^2^	29.4 ± 4.7	29.0 ± 4.2	29.7 ± 5.2	0.673	30.9 ± 6.1	30.5 ± 5.4	31.2 ± 6.1
Female, n (%)	41 (53.9)	21 (50.0)	20 (58.8)	0.443	56 (45.2)	27 (46.6)	28 (42.4)
Race, n (%)				0.199			
White	71 (93.4)	41 (97.6)	30 (88.2)		112 (90.3)	52 (89.6)	60 (91.0)
African American	0 (0.0)	0 (0.0)	0 (0.0)		5 (4.0)	3 (5.2)	2 (3.0)
Asian	1 (1.3)	0 (0.0)	1 (2.9)		1 (0.8)	0 (0.0)	1 (1.5)
American Indian or Alaska Native	0 (0.0)	0 (0.0)	0 (0.0)		1 (0.8)	0 (0.0)	1 (1.5)
Other/unknown	4 (5.3)	1 (2.4)	3 (8.8)		5 (4.0)	3 (5.2)	2 (3.0)
Ethnicity, n (%)				0.211			
Hispanic	4 (5.3)	1 (2.4)	3 (8.8)		1 (0.8)	1 (1.7)	0 (0.0)
Non-Hispanic	72 (94.7)	41 (97.6)	31 (91.2)		123 (99.2)	57 (98.3)	66 (100.0)
Education, n (%)				0.210			
High school degree	32 (42.1)	15 (35.7)	17 (50.0)		42 (33.9)	22 (37.9)	20 (30.3)
College degree or higher	44 (57.9)	27 (64.3)	17 (50.0)		66 (53.2)	29 (50.0)	37 (56.0)
Did not respond	0 (0.0)	0 (0.0)	0 (0.0)		16 (12.9)	7 (12.1)	9 (13.6)
ASA physical status, n (%)				0.000			
< 3	19 (25.0)	5 (11.9)	14 (41.2)		25 (20.2)	3 (5.2)	22 (33.3)
≥ 3	49 (64.5)	35 (83.3)	14 (41.2)		88 (71.0)	50 (86.2)	38 (57.6)
Not available	8 (10.5)	2 (4.8)	6 (17.6)		11 (8.8)	5 (8.6)	6 (9.1)
MiniCog, n (%)				0.091			
≤ 2	10 (13.2)	8 (19.0)	2 (5.9)		16 (12.9)	11 (19.0)	5 (7.6)
≥ 3	66 (86.8)	34 (81.0)	32 (94.1)		102 (82.2)	44 (75.9)	58 (87.9)
Not available	0 (0.0)	0 (0.0)	0 (0.0)		6 (4.8)	3 (5.2)	3 (4.5)
Verbal fluency, n (%)				0.163			
≤ 16	19 (25.0)	13 (31.0)	6 (17.6)		45 (36.3)	31 (53.4)	14 (21.2)
≥ 17	56 (73.7)	28 (66.6)	28 (82.4)		78 (62.9)	27 (46.6)	51 (77.2)
Not available	1 (1.3)	1 (2.4)	0 (0.0)		1 (0.8)	0 (0.0)	1 (1.5)
FRAIL, n (%)				0.371			
Robust	21 (27.6)	9 (21.4)	12 (35.3)		35 (28.2)	9 (15.5)	26 (39.4)
Pre-frail	42 (55.3)	24 (57.1)	18 (52.9)		60 (48.4)	31 (53.4)	29 (43.9)
Frail	12 (15.8)	8 (19.0)	4 (11.8)		20 (16.1)	15 (25.9)	5 (7.6)
Not available	1 (1.3)	1 (2.4)	0 (0.0)		9 (7.3)	3 (5.2)	6 (9.1)
Invasiveness, n (%)				0.008			
Tier 1 or 2	48 (63.2)	21 (50.0)	27 (79.4)		77 (62.1)	32 (55.1)	45 (68.2)
Tier 3 or 4	28 (36.8)	21 (50.0)	7 (20.6)		46 (37.1)	25 (43.1)	21 (31.8)
Not available	0 (0.0)	0 (0.0)	0 (0.0)		1 (0.8)	1 (1.7)	0 (0.0)
Surgery duration, min	170.8 ± 90.1	164.7 ± 73.0	177.3 ± 108.9	0.549	185.5 ± 147.8	168.5 ± 104.0	199.0 ± 174.6
LOS, days				0.028			
Median (Q1, Q3)	4 (2, 5)	4 (3, 6)	2 (2, 4)		3 (2, 4)	4 (2, 5)	2 (2, 4)
6-month mortality	0	0	0		0	0	0
Postoperative Delirium, n (%)	22 (28.9)	14 (33.3)	8 (23.5)	0.349	12 (10.8)	6 (12.0)	6 (9.8)

*LOS* length of stay, *ASA* American Society of Anesthesiologists.

**Table 2:** Characteristics of delirious vs. non-delirious patients. p-values represent a comparison between delirious and non-delirious patients within each age group.

Characteristic	Older			Middle-aged		
	Delirium (N = 14)	No delirium (N = 28)	P-value	Delirium (N = 8)	No delirium (N = 26)	P-value
Age, years	78 ± 4	75 ± 4	0.027	57 ± 4	56 ± 3	0.452
Weight, kg	82.1 ± 16.5	79.2 ± 13.1	0.540	82.1 ± 11.2	84.9 ± 20.3	0.713
Body Mass Index, kg/m^2^	28.7 ± 4.5	29.0 ± 4.0	0.827	28.0 ± 4.5	30.2 ± 5.3	0.297
Female, n (%)	5 (35.7)	16 (57.1)	0.190	6 (75.0)	14 (53.8)	0.288
Race, n (%)			0.646			0.700
White	14 (100.0)	27 (96.4)		7 (87.5)	23 (88.5)	
African American	0 (0.0)	0 (0.0)		0	0	
Asian	0 (0.0)	0 (0.0)		0	1 (3.8)	
American Indian or Alaska Native	0 (0.0)	0 (0.0)		0	0	
Other/unknown	0 (0.0)	1 (3.6)		1 (12.5)	2 (7.7)	
Ethnicity, n (%)			0.646			0.675
Hispanic	0 (0.0)	1 (3.6)		1 (12.5)	2 (7.7)	
Non-Hispanic	14 (100.0)	27 (96.4)		7 (87.5)	24 (92.3)	
Education, n (%)			0.495			0.106
High school degree	6 (42.9)	9 (32.1)		6 (75.0)	11 (42.3)	
College degree or higher	8 (57.1)	19 (67.9)		2 (25.0)	15 (57.7)	
ASA physical status, n (%)			0.717			0.823
< 3	1 (7.1)	4 (14.3)		3 (37.5)	11 (42.3)	
≥ 3	12 (85.7)	23 (82.1)		4 (50.0)	10 (38.5)	
Not available	1 (7.1)	1 (3.6)		1 (12.5)	5 (19.2)	
MiniCog, n (%)			0.052			0.752
≤ 2	5 (35.7)	3 (10.7)		0 (0.0)	2 (7.7)	
≥ 3	9 (64.3)	25 (89.3)		8 (100.0)	24 (92.3)	
Verbal fluency, n (%)			0.038			0.532
≤ 16	7 (50.0)	6 (21.4)		2 (25.0)	4 (15.4)	
≥ 17	6 (42.9)	22 (78.6)		6 (75.0)	22 (84.6)	
Not available	1 (7.1)	0 (0.0)		0 (0.0)	0 (0.0)	
FRAIL, n (%)			0.323			0.194
Robust	1 (7.1)	8 (28.6)		1 (12.5)	11 (42.3)	
Pre-frail	9 (64.3)	15 (53.6)		5 (62.5)	13 (50.0)	
Frail	3 (21.4)	5 (17.9)		2 (25.0)	2 (7.7)	
Not available	1 (7.1)	0 (0.0)		0 (0.0)	0 (0.0)	
Invasiveness, n (%)			0.513			0.176
Tier 1 or 2	6 (42.9)	15 (53.6)		5 (62.5)	22 (84.6)	
Tier 3 or 4	8 (57.1)	13 (46.4)		3 (37.5)	4 (15.4)	
Surgery duration, min	177.5 ± 58.3	158.3 ± 79.5	0.428	233.9 ± 117.1	159.9 ± 101.6	0.092
LOS, days			0.000			0.051
Median (Q1, Q3)	5.5 (5, 6.75)	4 (2.75, 4.25)		4 (2.75, 5.75)	2 (2, 4)	
6-month mortality rate	0	0		0	0	

*LOS* length of stay, *ASA* American Society of Anesthesiologists.

### Delirium-associated plasma protein expression is age-dependent

**Figure 1:**
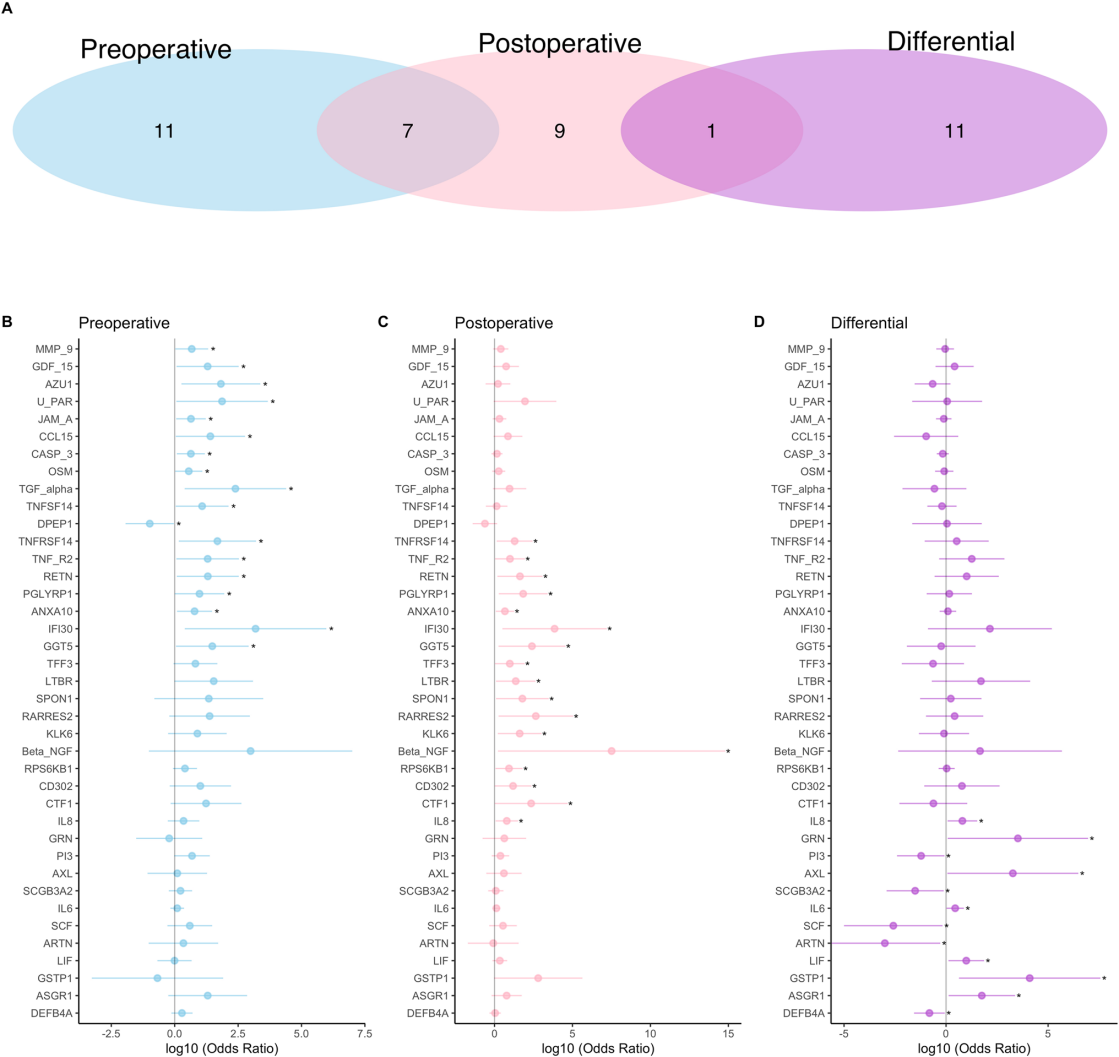
Delirium-associated plasma proteins in middle-aged patients. (**A**) Venn diagrams illustrate the plasma proteins significantly associated with postoperative delirium preoperatively (N = 18, blue), postoperatively (N = 17, pink), or differentially (N = 12, purple). (**B**–**D**) Forest plots for delirium-associated plasma proteins preoperatively, postoperatively, and by pre- to postoperative change. * denotes statistically significant proteins within each plot.

**Figure 2:**
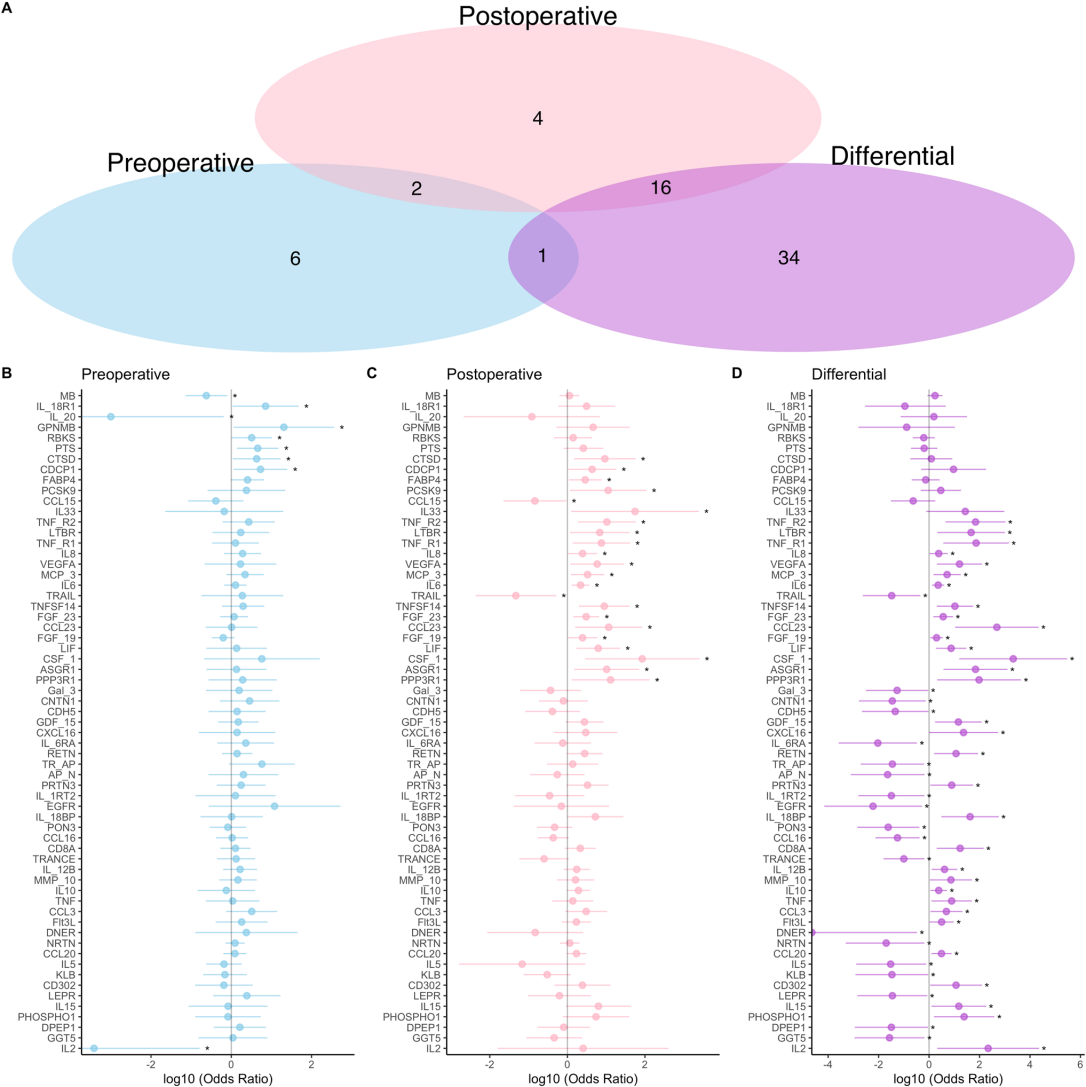
Delirium-associated plasma proteins in older patients. (**A**) Venn diagrams illustrate the plasma proteins significantly associated with postoperative delirium preoperatively (N = 9 blue), postoperatively (N = 22, pink), or differentially (N = 51, purple). (**B**–**D**) Forest plots for delirium-associated plasma proteins preoperatively, postoperatively, and by pre- to postoperative change. * denotes statistically significant proteins within each plot.

The plasma proteins associated with postoperative delirium varied with age- and perioperative time-point. In middle-aged subjects, 18 (6·7%) and 17 (6·3%) proteins in pre- and postoperative plasma, respectively, were associated with postoperative delirium (P < 0·05; Fig. 1). For all but one (DPEP1; OR < 1), delirium was associated with increased expression (OR > 1). In the older subjects, 9 (3·3%) and 22 (8·1%) proteins in pre- and postoperative plasma, respectively, were associated with postoperative delirium (Fig. 2). Again, delirium was associated mostly with higher expression, but the inverse was true preoperatively for myoglobin (MB), IL-2, and IL-20, and postoperatively for chemokine ligand 15 (CCL15) and TNF-related apoptosis-inducing ligand (TRAIL). None of the delirium-associated proteins in preoperative plasma were the same in both age groups, whereas in postoperative samples the age groups had 3 such proteins (IL-8, lymphotoxin beta receptor [LTBR], tumor necrosis factor receptor 2 [TNFR2]) in common.

Analysis of differential expression (i.e., preoperative to postoperative change) revealed 12 and 51 delirium-associated proteins in the middle-aged and older subjects, respectively (Figs. 1D and 2D), with expression of 7 (58·3%) in the middle-aged and 32 (62·7%) in the older group being increased. Only four proteins were associated with delirium in both age groups: IL-8, IL-6, leukemia inhibitory factor (LIF), and asialoglycoprotein receptor 1 (ASGR1).

### Gene set enrichment and protein–protein interaction analyses of delirium-associated proteins

**Figure 3:**
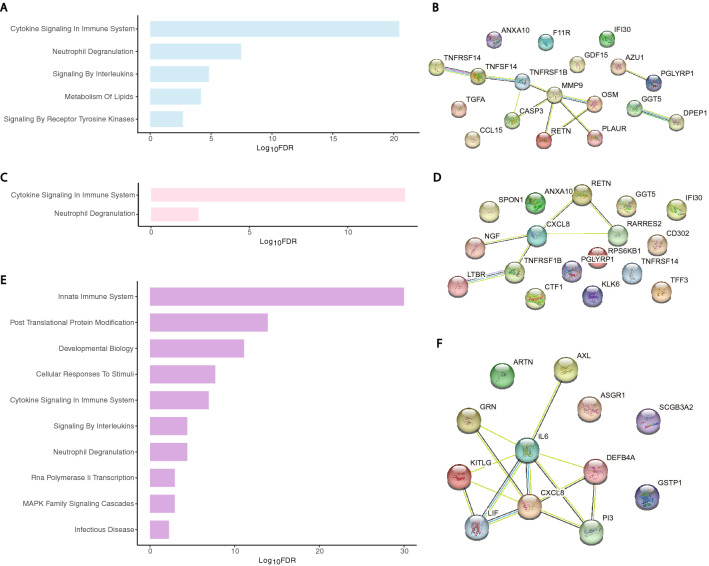
Functional analysis of delirium-associated proteins in plasma of middle-aged patients by GSEA and PPI network analysis. (**A**) Bar-plots of REACTOME gene sets significantly enriched in delirium-associated proteins in preoperative plasma (5 gene sets; blue bars). (**B**) Constructed PPI network for the preoperative time point (18 nodes and 11 edges, PPI enrichment < 10^−4^). (**C**) Bar-plots of REACTOME gene sets significantly enriched in delirium-associated proteins in postoperative plasma (2 gene sets; pink bars). (**D**) Constructed PPI network for the postoperative time point (17 nodes and 6 edges, PPI enrichment < 10^−2^). (**E**) Bar-plots of REACTOME gene sets significantly enriched in delirium-associated proteins by pre- to postoperative change (11 gene sets; purple bars). (**F**) Constructed PPI network for pre- to postoperative change (12 nodes and 14 edges, PPI enrichment < 10^−6^). Individual proteins in PPI networks are represented as nodes and interactions denoted by lines. For visual clarity, bar plots show a maximum of 10 of the most enriched pathways.

**Figure 4:**
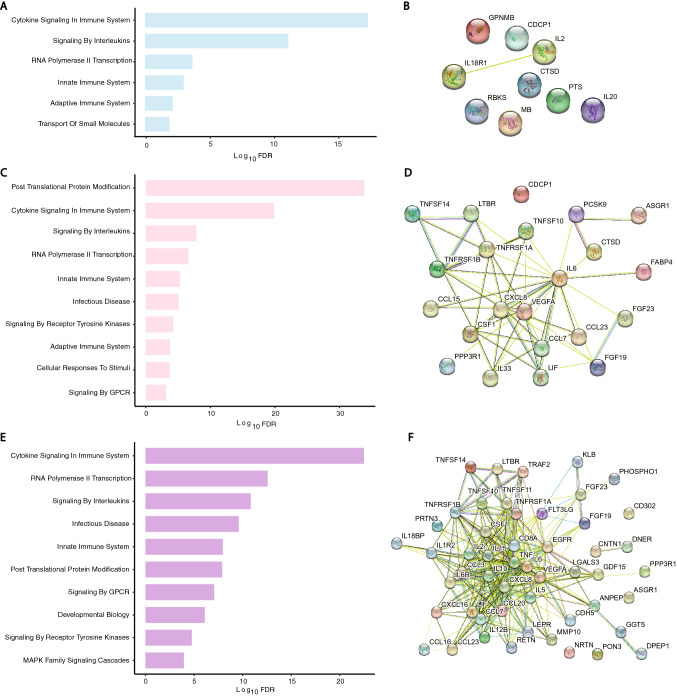
Functional analysis of delirium-associated proteins in plasma of older patients by GSEA and PPI network analysis. (**A**) Bar-plots of REACTOME gene sets significantly enriched for delirium-associated proteins in preoperative plasma (6 gene sets; blue bars). (**B**) Constructed PPI network for the preoperative time point (9 nodes and 1 edge) was not significant (PPI enrichment = 0.38). (**C**) Bar-plots of REACTOME gene sets significantly enriched for delirium-associated proteins in postoperative plasma (12 gene sets; pink bars). (**D**) Constructed PPI network for the postoperative time point (22 nodes and 56 edges, PPI enrichment < 10^−16^). (**E**) Bar-plots of REACTOME gene sets significantly enriched in delirium-associated proteins for pre- to postoperative change (16 gene sets; purple bars). (**F**) Constructed PPI network for pre- to postoperative change (51 nodes and 317 edges, PPI enrichment < 10^−16^). Individual proteins in PPI networks are represented as nodes and interactions denoted by lines. For visual clarity, bar plots show a maximum of 10 of the most significantly enriched pathways.

For GSEA and PPI analyses, timepoint-specific delirium-associated proteins were analyzed together irrespective of direction of change because there were too few proteins with OR < 1 for independent analyses. At the preoperative timepoint, there were five delirium-associated gene sets in the middle-aged group (FDR < 0.05; Fig. 3A) and six in the older group (FDR < 0.05; Fig. 4A). The middle-aged PPI network at the preoperative timepoint had 18 nodes and 11 edges (PPI enrichment < 10^−4^, Fig. 3B), but the PPI network for the older adults was not significant (PPI enrichment = 0.38; Fig. 4B).

At the postoperative timepoint, there were two delirium-associated gene sets in the middle-aged group and thirteen in the older group (Figs. 3C and 4C), with PPIs of 17 nodes and 6 edges (PPI enrichment < 10^−2^, Fig. 3D) and 22 nodes and 56 edges (PPI enrichment < 10^−16^, Fig. 4D), respectively. Inflammation-related gene sets were prominent in both ages, but additional networks were active in the older group including posttranslational protein modification and RNA polymerase II transcription.

**Figure 5:**
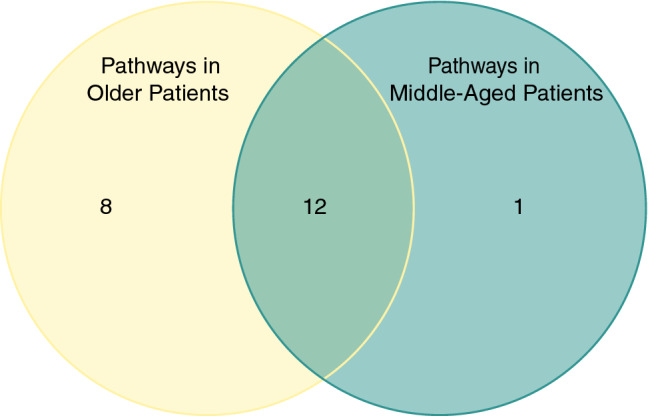
Venn diagram of delirium-associated gene sets, irrespective of time point. Among the protein sets of the different age groups, there were 8 REACTOME gene sets uniquely enriched in the old and one in the middle-aged. Twelve gene sets were common to both age groups.

By pre- to postoperative change in expression, there were 11 delirium-associated gene sets in the middle-aged and 16 in the older group (Figs. 3E and 4E). Notably, all sets but one in the former were enriched in the latter. The PPI network for the middle-aged group had 12 nodes and 14 edges (PPI enrichment < 10^−6^, Fig. 3F), whereas that of the older group had 51 nodes and 317 edges (PPI enrichment < 10^−16^, Fig. 4F). We also compared enriched gene sets across age groups regardless of time point (aged cohort = 20 gene sets total; middle-aged = 13) and identified twelve delirium-associated sets common to both age groups (Fig. 5). There was one gene set unique to the middle-aged group, “vesicle-mediated transport,” while those unique to the older-group included several related to GPCR (3 sets) and second messenger (2 sets) signaling.

### Plasma proteins and protein networks associated with delirium irrespective of age

We also analyzed the proteomic data for the combined group of 76 patients while controlling for age, sex, and years of education. This revealed 9 delirium-associated proteins preoperatively and 31 postoperatively (Supplementary Fig. 2). For 8 of 9 proteins preoperatively and 28 of 31 postoperatively, higher expression was associated with increased odds of delirium; the remainder were inversely associated. GSEA revealed 14 delirium-associated gene sets preoperatively and a PPI network with 9 nodes and 4 edges (PPI enrichment < 10^−2^, Supplementary Fig. 3) whereas there were 14 gene sets postoperatively and a PPI network of 31 nodes and 124 edges (PPI enrichment < 10^−16^, Supplementary Fig. 3). Analyzing pre- to postoperative change in expression yielded 45 delirium-associated plasma proteins, with increased expression of 33 (73%) and decreased expression of 12 (27%) conferring greater odds of postoperative delirium (Supplementary Fig. 2). GSEA identified 15 enriched gene sets and a PPI network of 45 nodes and 240 edges (PPI enrichment < 10^−16^, Supplementary Fig. 3).

## Discussion

This study compared part of the plasma proteome of postoperative delirium in two age groups and reveals both similarities and differences in the delirium signature with age. Consistent with previous results, the plasma proteome of delirium in older patients was complex and included numerous proteins and pathways, mostly related to inflammation and immune regulation. In contrast, the proteomic signature of delirium in middle-aged subjects was less intricate and included only a relatively small number of the proteins and pathways enriched in the older patients, though both fit the delirium phenotype. In fact, only seven plasma proteins and twelve protein networks were common to delirium in both ages. Notable among these are IL-6 and IL-8, which have been linked to delirium in older surgical patients and been proposed as potential biomarkers for it in several prior studies^12,14,27,28^. Our results indicate those associations are accurate irrespective of age and suggest that these and the other age-independent delirium-associated plasma proteins and pathways reflect core pathophysiological features of the syndrome and could be universal, age-independent plasma biomarkers for it. On the other hand, the proteins and pathways unique to delirium of older age may reflect, and potentially contribute to, the greater vulnerability and/or morbidity of the syndrome in older persons. Either way, our results reveal age differences in the plasma proteome of this common postoperative syndrome.

Overall, 89 (33%) of the 273 proteins on the panel were associated with postoperative delirium at some point in the perioperative period in one or both age groups and this signature was dynamic. Postoperative plasma and pre- to postoperative concentration changes were most revealing, as only a minority of differences were evident in the preoperative plasma of delirious subjects, and then only in the younger cohort. This suggests the search for predictive biomarkers of delirium may be more fruitful in middle-aged than older surgical patients. Of the 89 delirium-associated proteins, some have been linked to the syndrome previously. Examples include IL-6 and IL-8^9,12,14,26,27,29^, albeit only in older subjects, and CHI3L1, which has recently been identified as a potential predictive and disease biomarker for delirium^13^ and was positive in our analysis of delirium cases irrespective of age. However, we also uncovered associations between delirium and plasma proteins not reported previously. Examples include IL-15, a proinflammatory cytokine implicated in the pathophysiology of Alzheimer’s Disease and frontotemporal dementia^30^; CCL23, a chemokine involved in brain injury-induced neuroinflammation and progressive cognitive impairment^31^; TNF receptors 1 and 2 (TNFR 1 and 2)^32^; and CSF-1, a molecule critical for activation of cerebral microglia and strongly implicated in neurodegenerative disease^33^. GSEA and PPI network analyses similarly demonstrate delirium-associated changes in a multitude of pathways, mainly involving cytokine, chemokine, and interleukin signaling but also a few unexpected networks. One example of the latter is metabolism of lipids, concentrations of which are altered in several neurologic diseases and in the cerebrospinal fluid of older patients postoperatively^34,35^. Expression of many of these proteins was decreased in the context of delirium, as observed previously^12,13^, suggesting that dysregulation or imbalance of the systemic inflammatory milieu, not just activation, is a pathophysiologic characteristic of the syndrome. Collectively, our findings are consistent with the concept that systemic inflammation is a fundamental driver of postoperative delirium, even in middle-aged persons, and add several new candidates to the growing list of proteins and pathways that may be involved in delirium susceptibility and pathogenesis. However, our results are associative in nature, so it is still unclear whether these molecules are causes or merely markers of delirium. Either way, age proves to be an important factor.

Delirious middle-aged and older surgical patients had remarkably few delirium-related proteins and networks in common. Among proteins, they had just three postoperatively (IL-8, LTBR, and TNF-R2) and four by pre- to postoperative change (IL-8, IL-6, LIF, and ASGR1) in common. IL-6 and IL-8, as mentioned, have been strongly associated with delirium in older subjects previously^12,14,27,28^. Our results demonstrate that association holds in middle-aged patients as well, thus strengthening the case that these proteins could be useful, age-independent biomarkers for delirium. None of the other proteins have been linked to delirium before, although TNFR1 (but not TNFR2) is implicated in the delirium of critical illness^36^. All are inflammation related. LTBR belongs to the TNF receptor superfamily and is enriched in myeloid cells and monocytes, which are key mediators of clinical outcomes of surgery^17,37^. There is little information about the regional or cellular specificity of LIF and ASGR1 in the human brain and both are poorly expressed there. LIF is a cytokine in the IL-6 family and a stem cell growth factor that is not detected in immune cells but reportedly has anti-inflammatory and neuroprotective properties^38,39^ and ASGR1, a transmembrane protein, is found mainly in myeloid cells and liver but has no known function in brain. Pathway analyses likewise revealed relatively little overlap between the ages. At the postoperative time point, one gene set was common to both age groups and by pre- to postoperative change there were nine. Confirmation is required but the association of these proteins and networks with delirium in both age groups implies they represent a core feature, and possibly an essential part, of the pathophysiology of the syndrome. As such, proteins common to the condition across age could be universal, age-independent biomarkers for the syndrome.

The differences in the delirium signature with age are also interesting. Overall, the profile is more complex in older subjects. By pre- to postoperative change, for instance, there were 12 delirium-associated proteins and 11 delirium-associated networks in middle-aged patients but 51 proteins and 16 networks in the older group. However, 9 of the networks were common to both age groups. Thus, it appears that the additional proteins and networks associated with delirium in older patients may not be essential for it since the same clinical phenotype occurs in younger surgical patients without them. One possibility is that the proteins and networks unique to the delirium of older age represent parallel processes or consequences of delirium, rather than a direct cause of it, and contribute in some still undefined way to the greater vulnerability and morbidity of delirium in older patients. Along these lines, older patients with delirium had worse preoperative cognition by MiniCog and verbal fluency than age-matched patients without delirium. Poor cognition is a well-established predisposing factor for delirium but, in and of itself, is unlikely to account for the biomarker changes we identified because about half of the delirious older patients with biomarker changes had normal cognition (64% by MiniCog, 43% by verbal fluency). Nonetheless, more work is necessary to understand how predisposing and/or precipitating factors for delirium influence its plasma biomarker profile.

This study has both strengths and limitations. Chief among the former is that we included both middle-aged and older patients undergoing similar surgical procedures and having the same clinical syndrome. Additionally, we utilized a high sensitivity assay and a large protein panel and analyzed results at both the level of individual proteins and functional networks. Limitations include that this was a single-center study of subjects with relatively high educational attainment and low racial diversity and that plasma does not necessarily reflect the status of the brain. As such, it is unclear whether these plasma proteins have an etiologic role in the cognitive dysfunction of delirium or are simply peripheral markers of the responsible CNS processes. Furthermore, our study focused on neurosurgical and orthopedic spine surgery, which improves sample homogeneity but limits generalizability. The higher delirium rate in the included versus excluded subjects raises the possibility of selection bias but this seems unlikely because exclusion was based solely on plasma sample availability, enrollment criteria and experimental procedures were otherwise identical, and there was no difference between included and excluded subjects in other demographic, health, and outcome measures. Another limitation is that our sample size for delirium was relatively small (N = 22), though it is similar to or larger than other recent studies of the delirium proteome^12,13,40^. We used a validated and widely-accepted combined method (3D-CAM and chart review) to identify delirium^13,41–43^ but no method is perfect and we could have missed some cases. Work-flow for the proteomic analysis required a 2nd freeze–thaw cycle after plate preparation; while this could introduce pre-analytic variation, all samples were processed identically so it is unlikely to affect within or between group comparisons. Due to the exploratory nature of the work, we did not correct for multiple comparisons in the analyses of individual proteins, gene sets, or protein–protein network interactions and, therefore, cannot exclude the possibility of a type 1 error. Others investigating the plasma proteome of delirium have taken the same approach and our results confirm some previous findings but validation in larger data sets is necessary. Another limitation is that we utilized a curated panel of proteins. Although extensive, our platform included fewer proteins and used an analytical method different from those used in other recent work. Consequently, we did not assay some proteins implicated previously in the development of postoperative delirium (e.g. CRP) and possibly missed others that might be affected because they underwent post-translational modifications (e.g. phosphorylation) that produced proteoforms our assay could not recognize or were not on the panel. There are also differences between our results and previous reports for a few proteins. For example, delirium has been linked to upregulation of CHI3L1^13^, a protein associated with aging and chronic immune disorders, and to an increase in NFL, a potential plasma protein marker of neuronal injury^28,43^. We found an increase in CHI3L1 by differential expression when the age groups were combined (Supplemental Fig. 2D) but it did not emerge when the age groups were analyzed separately, possibly due to the smaller sample sizes in each age group. NFL was not a positive biomarker for delirium in any of our analyses. We are not unique in this regard^44,45^ and nor do all report a relationship between plasma NFL and postoperative cognitive outcomes^46^ but the different results could be explained by variations in the type and invasiveness of surgical procedures (cardiac vs. joint replacement vs. spine surgery), assay method (SOMAscan vs. proximity extension assay), or duration of postoperative follow-up. The latter is relevant because we collected our final plasma sample on postoperative day 1 and changes could have been missed because NFL peaks later.

In summary, we demonstrate that the plasma protein signature of postoperative delirium is age dependent. Some proteins and pathways, including plasma IL-6 and IL-8, are common to delirium in both age groups but older delirious patients had changes in more proteins and pathways than middle-aged subjects with the same clinical phenotype. This implies that there are age-related differences in the pathogenesis of delirium and/or its clinical consequences but mechanistic details are not clear. We speculate that the proteins and networks common to delirium in both ages reflect core pathogenic attributes of the condition, and could be age-independent biomarkers for it, whereas those unique to older delirious subjects may represent the greater susceptibility and/or morbidity of the syndrome in that age group. Additional studies of delirium in patients of different age are needed to validate these results and to distinguish the plasma signature of delirium per se from secondary but important and age-related aspects of this complex syndrome.

## Methods

### Study overview

This prospective observational study was approved by the Institutional Review Board of Partners Healthcare (now MassGeneralBrigham, Inc.) and all aspects of the study were performed in accordance with relevant guidelines and regulations. Patients were enrolled between November 19, 2018 and August 28, 2019 in the Cognitive Outcomes of Geriatric Surgery (COGS) Study, an ongoing investigation of contributors to and outcomes of postoperative delirium in patients aged 45–60 (middle-age) or ≥ 70 (older) years having elective spine surgery at Brigham and Women’s Hospital (BWH; Boston, MA). Details of COGS have been reported previously^42^ and recruitment and retention details are shown in a flow chart (Supplementary Fig. 1). In brief, we included patients aged 45–60 or ≥ 70 years of age with an ASA physical status of I-III presenting for elective spine surgery and excluded those with a history of stroke or brain tumor; vision or hearing impairment that would impair ability to see pictures or read/hear instructions); limited use of the dominant hand (impaired drawing ability); or inability to speak, read, or understand English. Other than a prior diagnosis of dementia, there were no exclusion criteria for education or cognitive function. Of the 278 eligible patients approached, 69 declined to participate and 9 were ineligible, for a recruitment rate of 72% (N = 200). Of those, 7 did not have surgery (2 middle-aged, 5 old), 6 unenrolled (3 middle-aged, 3 old), and 105 had insufficient plasma in the biorepository at one or both timepoints. Finally, 6 patients were excluded due to missing data on necessary covariates, leaving 76 subjects (N = 34 middle-aged, 42 older) with preoperative and postoperative samples for the proteomic analysis.

After obtaining written informed consent, study staff collected baseline demographic information (age, sex, BMI, highest level of education) from the electronic medical record (EMR) preoperatively and administered the MiniCog, animal verbal fluency tests, and FRAIL scale^2,42^. Delirium was identified by daily assessment with the 3-min diagnostic interview for CAM-defined delirium (3D-CAM)^52^ on postoperative days 1 to 3 and by chart review using published criteria^41,42^. We used both methods because they are complimentary and well-established.

Delirium waxes and wanes so the 3D-CAM will miss delirium if it occurs at other times, whereas chart review reflects events over an entire day but may miss hypoactive delirium. The sensitivity and specificity of the combined method is superior to that of either method alone and maximizes identification of delirium^41^. As such, it is commonly used^13,42,43^. The 3D-CM was administered in the patient’s room once per day in the morning prior to obtaining postoperative blood samples. Nearly all 3D-CAM assessments were performed by the same person (EJK), who was trained by a board-certified, clinically active geriatrician and was blinded to chart review information at the time of delirium screening. Patients were considered delirious if either the 3D-CAM or comprehensive patient chart review were positive^47^. Surgical invasiveness, hospital length of stay, and 6-month mortality were collected by systematic chart review or examination of discharge diagnoses in the BWH Research Patient Data Registry. Invasiveness was categorized based on a scale of 1 to 4, where Tier 1 is least invasive (e.g. microdiscectomy) and 4 is most invasive (e.g. tumor, deformity, combined anterior and posterior procedures)^3,48^. Data were collected and managed using Research Electronic Data Capture (REDCap), hosted at Partners Healthcare (Somerville, MA).

### Sample collection for protein analysis

Preoperative blood samples were collected with routine laboratory samples. If routine laboratory samples were not collected, a preoperative sample was collected on the day of surgery prior to entering the operating room. Postoperative samples were drawn by study staff on postoperative day one shortly after delirium testing. The scheduled time of surgery varied but most were morning or mid-day cases; because postop blood draws were done in the morning on postop day 1, the interval between surgery and follow up was 24 ± 4 h in the vast majority. Samples were collected via venipuncture using a 21-gauge Vacutainer needle and deposited into two 10 ml K2 EDTA anticoagulant tubes and processed within 30 min. To collect plasma, EDTA tubes containing whole blood were centrifuged at 2000 RPM (693 g) at room temperature for 10 min. Plasma was removed, aliquoted into a new tube, and centrifuged at 4000 RPM (2773*g*) at room temperature for 10 min, and the plasma was removed without disrupting the pellet, aliquoted, and frozen at − 80 °C until further use.

### Plate preparation

Plasma samples were thawed to room temperature, briefly vortexed, and then 20 µl of plasma per timepoint per patient was plated onto a AB-0800 0·2 ml Skirted 96-well PCR plate (Thermo Scientific). Each plate (N = 2) included both pre- and postoperative samples from a subset of patients, chosen by a list randomizer, and was run once; samples from four patients were included on both plates to control for batch effects. Plated samples were refrozen on dry ice and transported to Olink Proteomics (Watertown, MA). All samples were treated identically, so potential pre-analytic variation due to a 2nd freeze–thaw cycle would be the same across samples and groups.

### Proximity extension assay (PEA)

Two hundred and seventy proteins were analyzed using the Olink^®^ Inflammation, Cardiovascular III, and Neuro-Exploratory panels (Olink Proteomics AB, Uppsala, Sweden). The PEA technology has been well-described and enables simultaneous analysis of 92 analytes per panel using 1 µl of each sample^49^. In brief, pairs of oligonucleotide-labeled antibody probes bind to their targeted protein, which produces pair-wise hybridization when the two probes come into close proximity. Addition of a DNA polymerase produces a proximity-dependent DNA polymerization event and generates a unique PCR target sequence. The resulting DNA sequence is subsequently detected and quantified using a microfluidic real-time PCR instrument (Biomark HD, Fluidigm). Data are then quality controlled and normalized using an internal extension control and an inter-plate control to adjust for intra- and inter-run variation. The final read-out is an arbitrary unit on a log2-scale where a high value corresponds to higher protein expression. Assay validation data are available on the manufacturer's website (http://www.olink.com).

### Statistical analysis

Older and middle-aged groups were compared on demographic, clinical, and perioperative characteristics using parametric (e.g., *t* tests, χ^2^) or non-parametric (e.g., Wilcoxon rank-sum) statistical methods as appropriate. The characteristics of patients that were included and excluded from analysis were also compared. We based our sample size on previous studies of large protein sets that showed numerous delirium-associated proteins using similar or smaller sample sizes^12,13,40^.

The association between protein level (continuous variable) and post-operative delirium was calculated by logistic regression adjusted for age (continuous), gender, and years of education. We performed separate analyses for (1) preoperative; (2) postoperative day 1; and (3) pre- to postoperative change in the 273 proteins. We expressed results as the log of base 10 odds ratios (OR), with OR > 1 indicating a direct relationship (higher expression associated with increased odds of delirium) and OR < 1 indicating an inverse relationship (lower expression associated with increased odds of delirium). P < 0.05 was considered statistically significant. Results were not adjusted for multiple comparisons because this was an exploratory analysis. We used SAS 9·4 for logistic regression analyses and RStudio (version 1·2·1335 for MacOS), with “ggplot2” and “VennDiagram” packages, for data visualizations.

### Gene set enrichment analysis (GSEA) and protein network construction

Chi square tests were performed to test for proteins statistically associated with delirium preoperatively, postoperatively, or differentially using the REACTOME Pathway Database (N = 1532 gene sets)^50^ in category C2 of the Broad Institute’s Molecular Signatures Database^51,52^. A threshold of one shared protein between the gene set and the 273 Olink proteins was used as a cutoff. A false discovery rate (FDR) of < 0.05 was used as the significance threshold. Protein sets that met significance criteria were entered into the Search Tool for the Retrieval of Interacting Genes/Proteins (v11·5) to analyze protein–protein interactions (PPI) and identify functional interactions^53^. P < 0·05 was considered statistically significant.

## Supplementary Information

Supplementary Figures.

## Data Availability

Deidentified participant data that underlie the results reported in this manuscript will be shared beginning 12 months and ending 36 months after article publication with researchers who submit a methodologically sound proposal to the senior authors. Data requestors will also be required to sign a data access agreement.
