# Supplementary material for: Age-dependent differences and similarities in the plasma proteomic signature of postoperative delirium
Source: Sci Rep. 2023 May 8;13:7431. doi: 10.1038/s41598-023-34447-7 (PMC10167206; doi:10.1038/s41598-023-34447-7)
Supplement: Supplementary file 1 — Supplementary Figures. [file 41598_2023_34447_MOESM1_ESM.pdf]

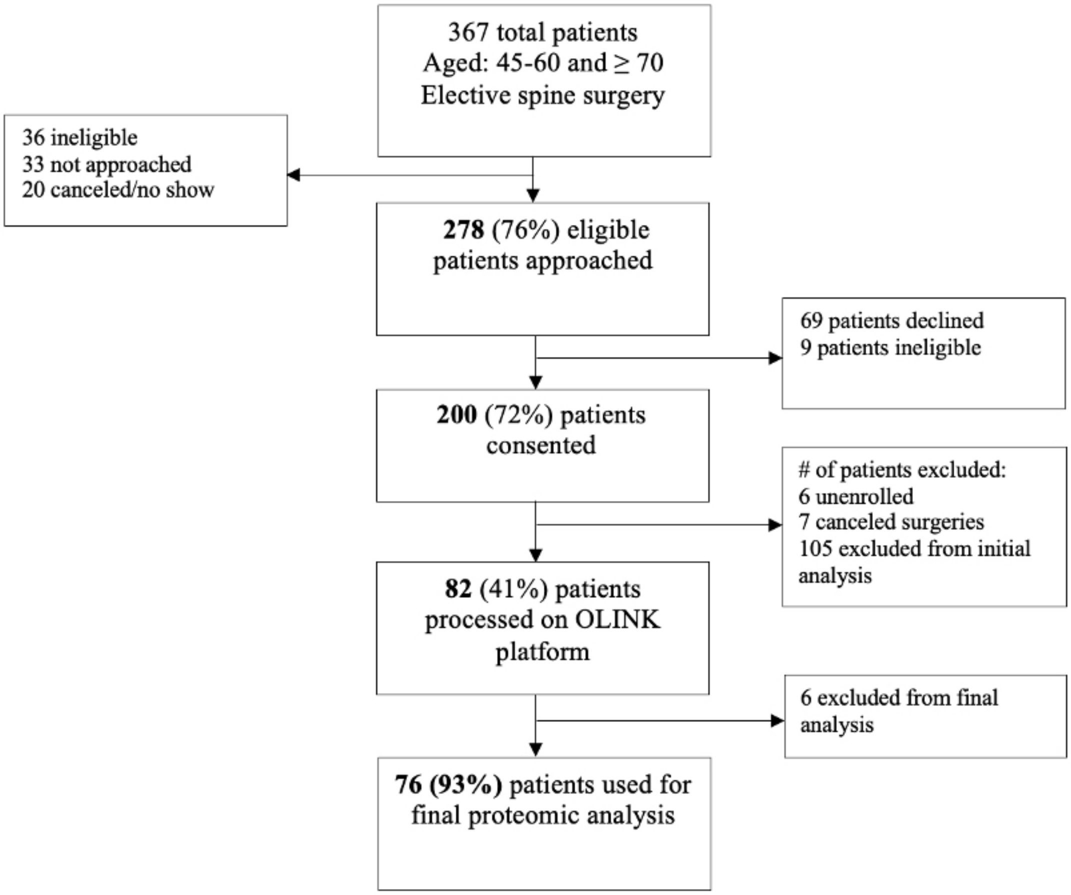

Supplemental Figure 1. Flow diagram of recruitment and retention.

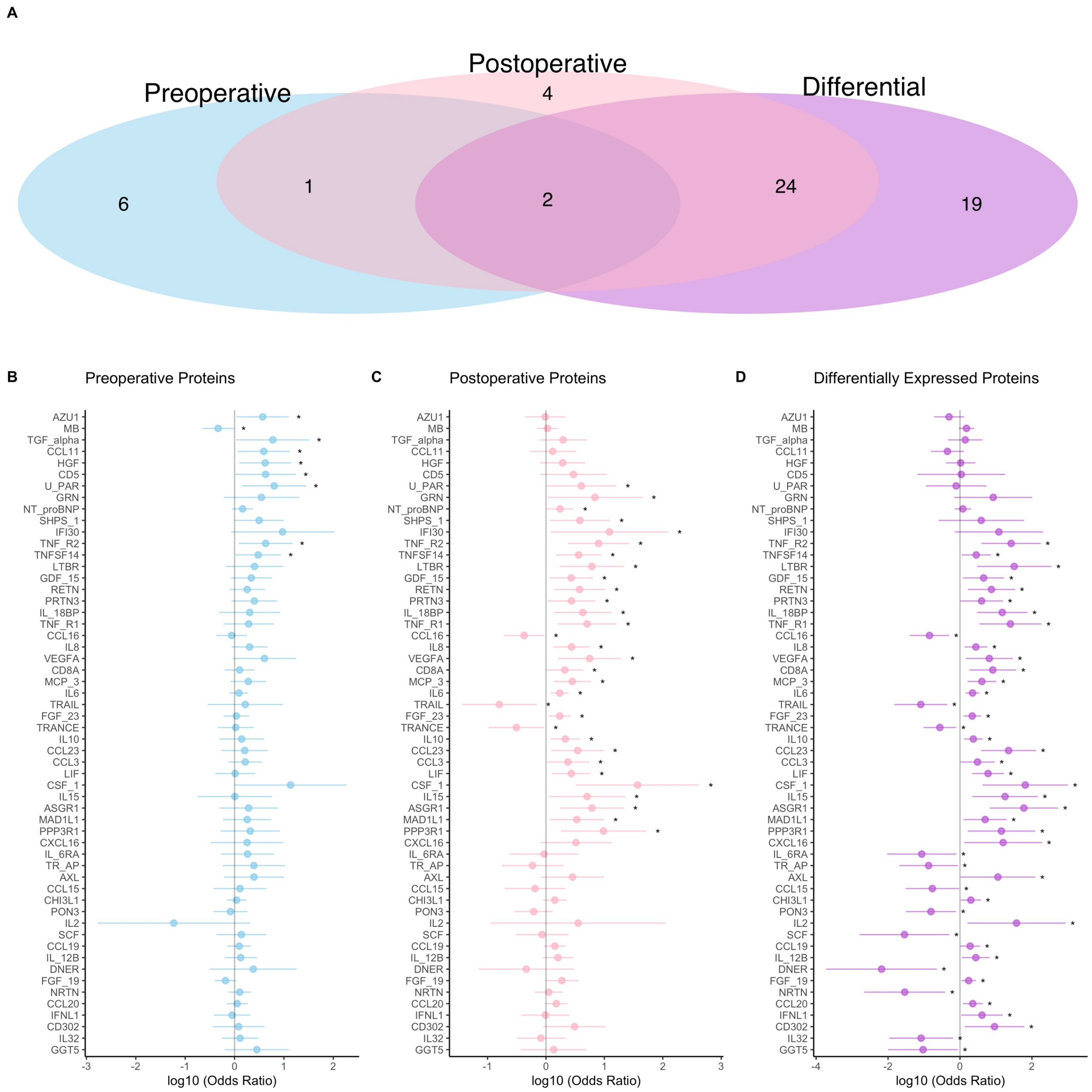

Supplemental Figure 2. Plasma proteins associated with postoperative delirium in spine surgery patients irrespective of age. Data were controlled for age, sex and years of education. (A) Venn diagrams illustrate the plasma proteins significantly associated with postoperative delirium preoperatively (n = 9 blue), postoperatively (n= 31, pink), or differentially (n = 45, purple). (B-D) Forest plots for delirium-associated plasma proteins preoperatively, postoperatively, and by pre-to postoperative change. * denotes statistically significant proteins within each plot.

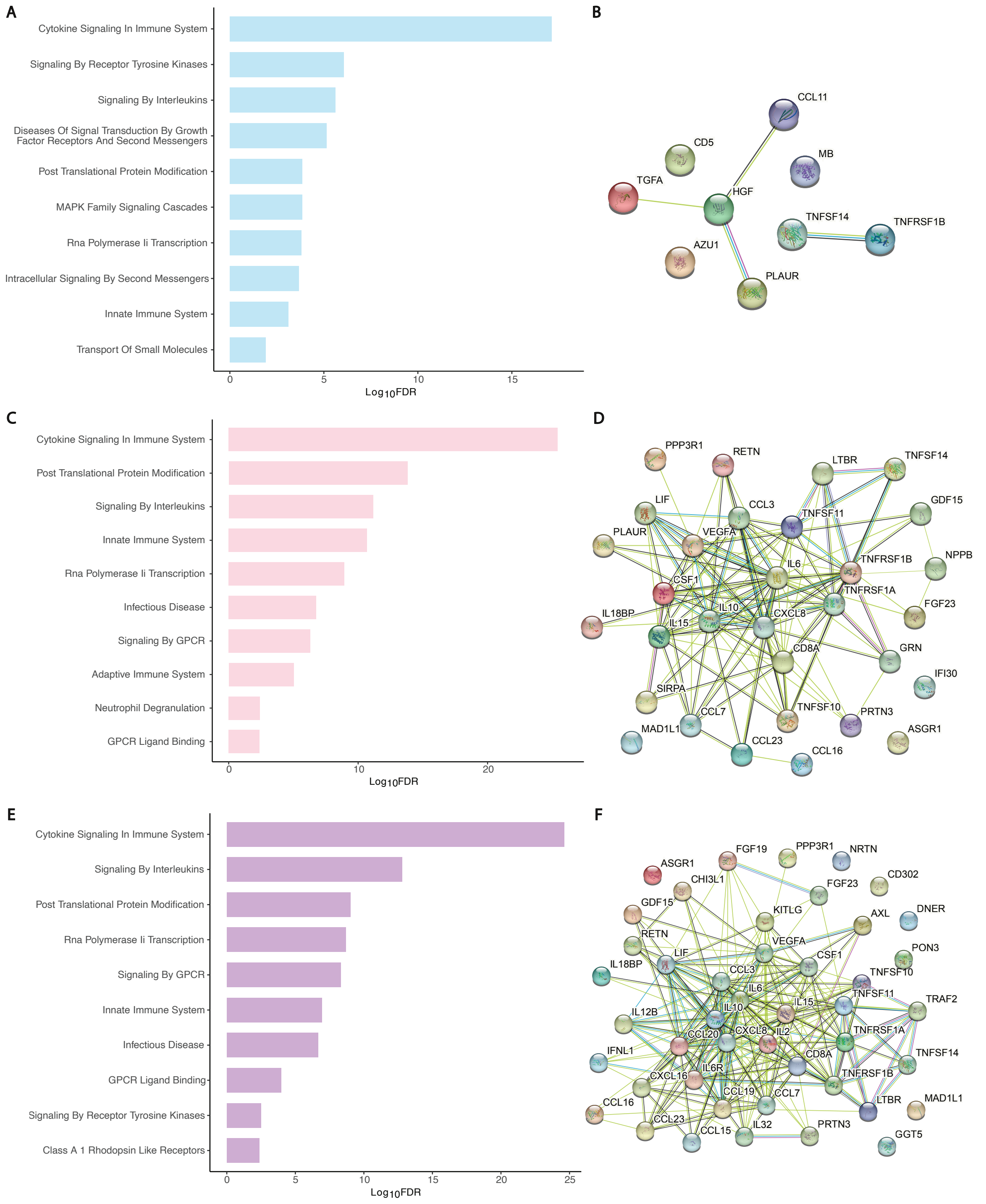

Supplemental Figure 3. GSEA and PPI network analysis of postoperative delirium-associated proteins in spine surgery patients irrespective of age. Data were controlled for age, sex and years of education. A, Bar-plots of REACTOME gene sets significantly enriched in delirium-associated proteins for preoperative period (14 gene sets; blue bars). B, Constructed PPI network for the preoperative time point (9 nodes & 4 edges, PPI enrichment < 10-2). C, Bar-plots of REACTOME gene sets significantly enriched in delirium-associated proteins for postoperative period (14 gene sets; pink bars). D, Constructed PPI network for the postoperative time point (31 nodes & 124 edges, PPI enrichment < 10-16). E., Bar-plots of REACTOME gene sets significantly enriched in delirium-associated proteins for pre- to postoperative change (15 gene sets; purple bars). F, Constructed PPI network for pre- to postoperative change (45 nodes & 240 edges, PPI enrichment < 10-16). Individual proteins in PPI networks are represented as nodes and interactions denoted by lines. For visual clarity, bar plots show a maximum of 12 of the most significant pathways.
